# Clinical-surgical treatment of temporomandibular joint disorder in a psoriatic arthritis patient

**DOI:** 10.1186/1746-160X-9-11

**Published:** 2013-04-04

**Authors:** Edela Puricelli, Adriana Corsetti, Julieta Gomes Tavares, Giuliano Henrique Mião Luchi

**Affiliations:** 1 Irmandade Santa Casa de Misericórdia de Porto Alegre, ISCMPA, Porto Alegre, RS, Brazil; 2 Faculdade de Odontologia, Pontifícia Universidade Católica de Porto Alegre, PUCRS, Porto Alegre, RS, Brazil; 3 Hospital de Aeronáutica de Canoas, HACO, Canoas, RS, Brazil

**Keywords:** Temporomandibular joint disorders, TMJ pain, Psoriasis, Mandibular condyle

## Abstract

**Introduction:**

Condylotomy is a surgical procedure that has been used as an option to treat temporomandibular disorder (TMD) patients. This technique has the advantage of avoiding intra-capsular alterations that might be found involving other surgical procedures. Its use, even when unilateral, has positive effect on treatment of both joints.

**Methods:**

In order to better evaluate the benefits of a clinical-surgical treatment for TMD, the present report describes the case of a psoriatic arthritis patient. The case was clinically characterized by dental malloclusion, and imaging exams showed joint degeneration of the right mandibular condyle. The patient was treated by condylotomy technique after a prosthetic oral rehabilitation.

**Results:**

No clinical-radiological signs or symptoms of progression of articular disease were observed within a period of 16 months after surgery. Furthermore, there was functional stability of the temporomandibular joint, total absence of local pain and improvement of mouth opening.

**Conclusion:**

The present study suggests that condylotomy can be considered as a valid option for the management of TMD, since it has low surgical morbidity and favorable clinical outcomes. In this case, the patient had a medical diagnosis of systemic disease presenting general pain and pain at the temporomandibular joint (TMJ), in addition of causal agent of TMD (dental malloclusion). The difficulty of finding a single etiology (malocclusion vs. systemic disease) did not exclude the indication of a clinical-surgical treatment to re-establish the balance of TMJ.

## Introduction

Currently, between 10% to 30% of the world’s population seeks specialized care for temporomandibular disorders (TMD) [1-3], which poses one of the most challenging treatment problems in the field of dentistry. Traditionally, conservative therapy is established by means of drugs, occlusal splints, occlusal adjustments, and oral rehabilitation. Invasive procedures are indicated when conservative therapies are not able to eliminate articular pain and restore functional jaw movements. Thus, surgical maxillofacial interventions may be a better option to treat patients in the presence of pathologies such as osteoarthritis, anterior disc displacement (with and without reduction) associated with untreatable joint pain and progressive degeneration of the mandibular condyle [3-6].

The classical method of closed condylotomy is indicated for the treatment of mandibular prognathism and is based on the Kostecka’s proposal, where a high vertical oblique osteotomy is performed bilaterally. According to Banks and Mackenzie [7], Ward, Smith and Sommar, in 1957, proposed for the first time a more vertical and less oblique condylotomy, indicating this modified procedure for TMD treatment. Other researchers, such as Nickerson and Veaco (1989) [1] and Upton and Sullivan (1991) [8], have suggested changes in this technique, so that it can be used in a broader context in the field of maxillofacial surgery. In addition, it has been a consensus that the gold standard procedure is unilateral condylotomy, performed first on the most involved TMJ [3,4]. To be effective, the condylotomy technique should include correct diagnosis and indication, preoperative procedures, and precise surgical planning. Also, the active or passive skeletal asymmetries that might interfere with the evolution and postoperative stability should be individually identified.

In relation to the surgical technique, Gerard suggested in 1996 metal fixation using the proximal instead of the distal segment reviewed in [3]. However, the original concept of mobility between the segments remains the first option because this procedure facilitates functional adaptation even in the presence of *osseous callus*[3]. The osteotomy is performed on the proximal area of the mandibular lingula followed by detachment of the medial pterygoid muscle up to 50% of its insertion. Afterwards, the muscle is inserted inferiorly in the proximal segment that contains the condyle, allowing the overlapping of the muscle over the residual mandibular ramus. Thus, there is a displacement of 3 to 5 mm of this segment assuming a more infero-posterior position, creating intra-articular spaces releasing the disc and bone-cartilaginous compression. The stabilization of fractured segments is accomplished by maxillomandibular elastic bands using orthodontic accessories, which are maintained for two to three weeks. After this period, a passive physical therapy is indicated along the presence of a stable dental occlusion. This treatment normally takes four months and is accomplished with a bilateral lightweight elastic traction that by the end of this period is only used at night [3,9].

Psoriatic arthritis (PA) is a chronic inflammatory disease associated with skin and/or nail psoriasis. The patients generally have negative rheumatoid factor (RF) and absence of rheumatoid nodules. The predominance of PA among individuals with skin psoriasis has varied between 2.6% and 7%. This disease has the potential to be extremely severe and results in important functional disability. The severity of signs and symptoms of skin psoriasis is correlated with the emergence of PA. The articular disease manifestations occur approximately 10 years after the first signs of skin psoriasis. PA belongs to a group of inflammatory arthritis called spondyloarthropathies and shares some clinical and laboratorial characteristics with other diseases within the same group [10], making the differential diagnosis more challenging. The clinical manifestations of PA rarely include TMJ symptoms, which is usually bilateral causing pain and limitation of jaw movement. The treatment of PA must be multidisciplinary involving general care and dental approaches. The use of occlusal devices, physiotherapy and intra-articular infiltration are described as efficient in controlling pain symptomatology [11,12]. In the present study, the authors propose that a prosthetic oral rehabilitation and a surgical intervention are efficient in reestablishing the functional balance of the TMJ.

## Case presentation

Patient MBC, female, 67 years old was sent to the Dental Clinical Center of Santa Casa de Misericórdia of Porto Alegre - RS - Brazil, presenting medical history of psoriatic arthritis. The patient signed an informed consent for publication of this case report and accompanying images. This study was approved by the institutional Ethics Committee Research, is in accordance with the Declaration of Helsinki and follows the guidelines from the Ministry of Health resolution 196/96.

### Clinical and radiographic assessment

**Figure 1:**
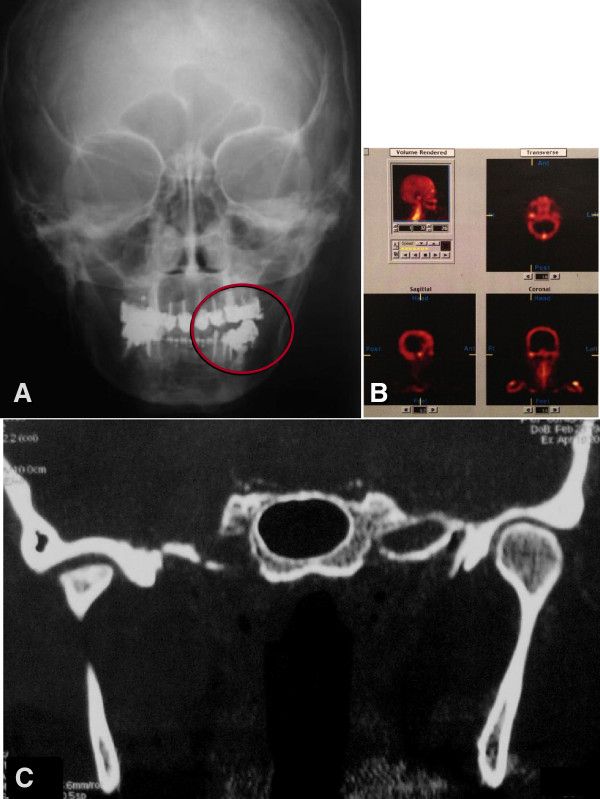
**Preoperative evaluation.** (**A**) Frontal initial pre-operative X-ray (frontal teleradiography): An open bite on the left side was present. (**B**) Bone scintigraphy: High bone metabolic activity was present at the right TMJ. (**C**) Computed Tomography in coronal sections of the face: Compared to the left side, the right TMJ presented a substantial loss of condyle anatomy associated with areas of bone sclerosis. The asymmetry between the mandibular ramus marks the lateral deviation of the jaw to the right side.

The patient complained about pain in the hands and feet articulations. In the right TMJ, the intensity of pain was 8 out of 10 on a visual analogue scale (VAS). At clinical examination, facial asymmetry, contralateral (left) posterior open bite (3 mm), lateral deviation of the jaw to the right side during habitual occlusion and mouth opening limitation (5 mm) were found. Imaging exams (panoramic radiography and computed tomography of TMJs) showed severe joint degeneration of the right mandibular condyle (Figure 1). Imaging techniques and clinical examination were used to establish a differential diagnosis and as a control in follow-up. The initial treatment was aimed at reducing the signs and symptoms (clinical pain and intra-articular inflammation).

### Initial clinical treatment

**Figure 2:**
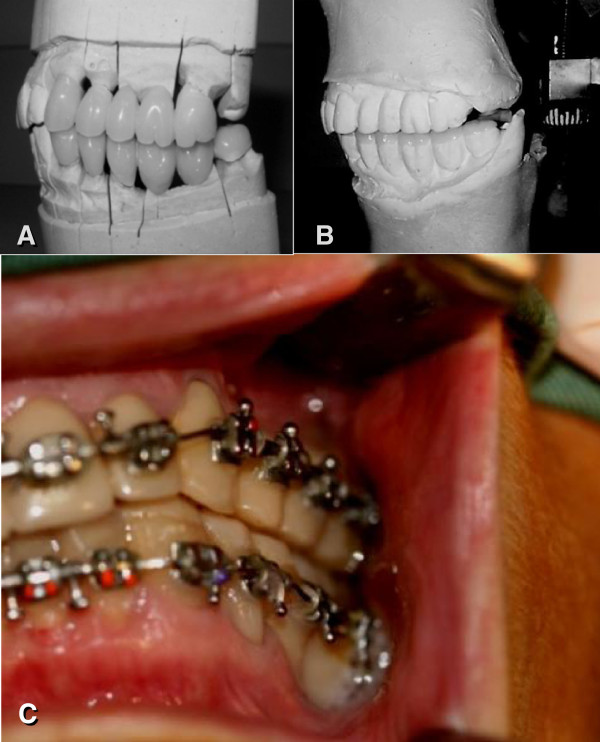
**Prosthodontic and orthodontic preparation.** (**A**) Initial prosthodontic preparation: Provisional prosthesis prepared prior to the surgery. (**B**) Provisional crowns made using pre-determined vertical dimension to provide the reposition of the right side of mandible after condylotomy. The occlusal transparent area demarcates the increase in final height of the crowns. (**C**) Orthodontic preparation: Pre-operative dental occlusion and fixation of orthodontic apparatuses with passive metal arches.

Provisional acrylic crowns that were adjusted using custom occlusal template and work models mounted on proper semi-adjustable articulator replaced unsatisfactory metallo-ceramic crowns on the left inferior-posterior teeth. Other occlusal parameters such as posterior vertical dimension, interocclusal tooth contacts on the working side, and avoidance of occlusal contacts on the balancing side were restored (Figure 2 A, B). Hard stabilization appliance was provided on inferior arch to reduce joint loading and deprogram central patterns that would maintain parafunctional oral habits. This device was used continually and only removed on main meals. The clinical evolution was followed up for 11 months and the follow-ups were scheduled every 45 days. Improvements to the patient’s condition such as increasing amplitude of mandibular movements and reduction of clinical pain levels to 4 out of 10 on the pain scale were observed in the first three months after the surgical procedure. Although the patient showed an improvement in the clinical conditions, CT images indicated progressive intra-articular disease. The posterior open bite was increased by another 3 mm after the first oral rehabilitation treatment.

### Surgical treatment

**Figure 3:**
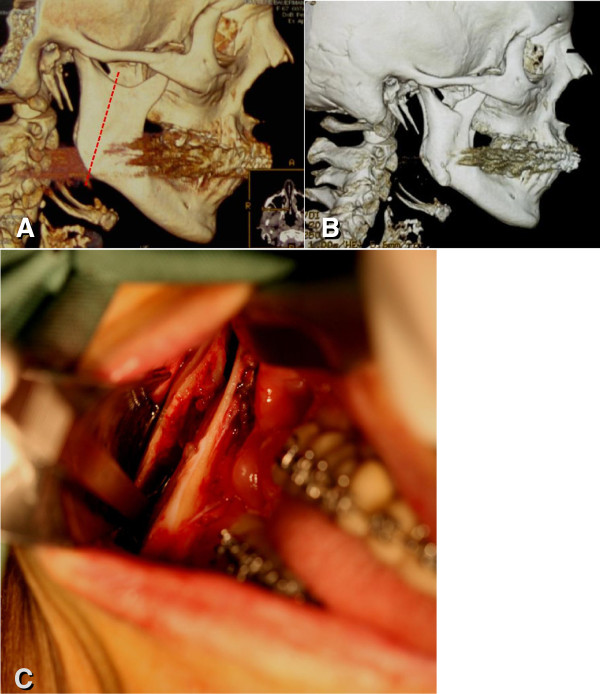
**Condylotomy.** (**A**) Schematic drawing of the osteotomy: Pre-operative 3D-CT image shows the right side of the patient. The planned osteotomy was mapped into a dotted line drawn on the image. (**B**) 3D-CT 12 months after the surgery: Functional reposition of the right condyle to an anterior position and consequent shortening in transverse extension of the mandibular incisure was observed. (**C**) Intra-oral view during surgery: The overlap of the posterior segment (ramus and condyle) over the remaining segment of the mandible was possible to be observed. This technique does not use metal fixation.

After clinical and imaging assessments, condylotomy was indicated as first-line treatment option to improve the patient’s condition. The preoperative preparation included new acrylic crowns to improve dental contacts and occlusion stability, and bonding of orthodontic accessories for the maxillomandibular block trans/post-surgical (Figure 2C). Under general anesthesia, an osteotomy was made on the proximal area of the mandibular lingula from the mandibular incisure to the mandibular angle (Figure 3). After osteotomy, orthodontic anchorages were used to perform an intermaxillary block using elastic bands. The correct location and stability of the overlapping mandibular segments were carefully observed. Then, the soft tissue flap was repositioned and sutured with isolated stitches.

### Post-surgical follow-ups

**Figure 4:**
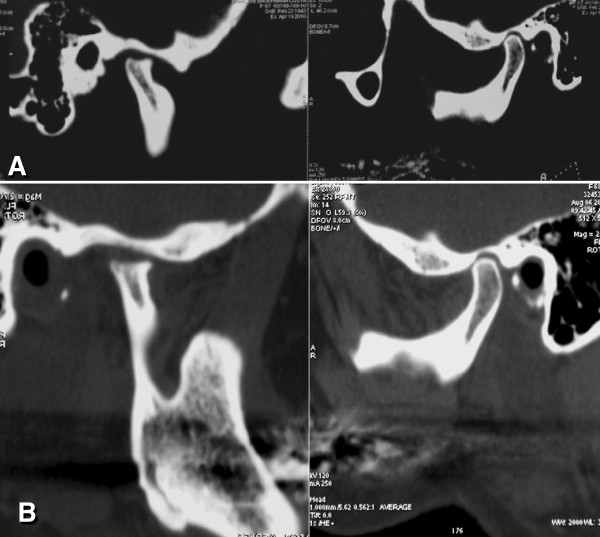
**Pre- and postoperative CTs.** (**A**) Pre-operative saggital-CT images (sections in closed-mouth position) suggested degenerative changes in right condyle. Left side showed no changes. (**B**) Post-operative sagittal CT: At post-operative period of 16 months, image showed no indication of progression of the degenerative changes in the right or left condyle.

After 6 months, the patient reported no pain and mouth opening of 36 mm. The orthodontic accessories were removed and the definitive prosthetic rehabilitation started. During the 12 months of follow-up, clinical and imaging exams demonstrated stable results with adequate mandible functions (42-mm mouth opening) and the patient reported no pain. After 16 months of follow-up, the patient remained stable, without clinical complaints. However, due to the systemic disease, the patient was advised to keep periodic medical and dental clinical controls (Figure 4).

## Discussion

In many cases, conservative treatments are able to recover a healthy condition of some patients with intra-articular diseases of TMJ but they might not be effective on others. In these cases, surgical procedures become options of treatment to restore masticatory function, reduce clinical pain [3,9,13], and decelerate reabsorption processes in the joint [3]. Thus, each case has to be individualized in terms of diagnosis, prognosis and therapeutic indication. CT images are an important tool during the diagnostic process due to high detection of degenerative process in the hard tissues of TMJ. Moreover, CT allows analyzing the effects of the applied therapy and comparisons before and after the treatment. In this particular case, the objective results found in CT images were able to confirm the stabilization in the condyle degeneration process. Moreover, these imaging findings were correlated to the improvement of clinical symptoms. Condylotomy has been suggested as the indicated treatment of TMDs in cases of internal disorders [14,15]. More than a simple guided mandibular fracture, condylotomy should be seen as an indirect arthroplasty, without direct invasion of joint structure. The possible benefits from condylotomy include increase of intra-articular space, decompression of the cartilaginous tissues and improvement of the condyle inclination allowing the recapture of the articular disc [3,16-18]. Studies by Puricelli [3] and Upton [19] emphasize the clinical improvement of patient’s condition and the low frequency of necessity of re-operations (common in other surgical approaches) associated with the condylotomy. Albury [16], in a retrospective study of 78 condylotomy surgeries in 63 patients, reported significant improvement on pain condition in 94% of patients. Hall and Werther [20] studied the necessity of re-operation after condylotomy of 361 joints in 235 patients, and reported only 4.4% of re-interventions. Subsequently the same group, using magnetic resonance, assessed the relationship of the articular disc in 80 symptomatic cases of disc displacement submitted to condylotomy and found disc reduction after the surgery in 79% of cases [21].

In the present clinical case, the patient had a medical diagnosis of systemic disease presenting general pain (especially on hands and feet) and pain at TMJ, in additional of causal agent of TMD (dental malloclusion). The difficulty of finding a single etiology (malocclusion vs. systemic disease) did not exclude the indication of a clinical-surgical treatment to re-establish the balance of TMJ.

At the time of the first appointment, in the presence of articular degenerative process and unstable occlusion, there were no conditions to predict the evolution of the left open bite. In this situation, it was impossible to start any definitive occlusal treatment. After the condylotomy, the articulated segment was released allowing functional adjustments and a new tridimensional skeletal relation, functionally assisted by a rehabilitated dental occlusion. The benefic effect of condylotomy to the patient was observed clinically. For example, the skeletal asymmetry that was found during preoperative evaluation was not present after the clinical-surgical treatment. Thus, a facial harmony including skeletal-muscle symmetry and balanced occlusion was reestablished in the patient’s stomatognathic system.

The present study suggests that this condylotomy can be considered as a valid option for the management of TMD because it has low surgical morbidity and favorable clinical outcomes. Moreover, condylotomy ensures a wide space in the joint, a desirable disk-to-condyle relationship, and decreases clinical pain. Ideally, this surgical procedure is indicated after the patient has been clinically treated and the dental occlusion has been reestablished. This condylotomy has unilateral indication, preferably applied to the most affected TMJ by both symptoms and bone alterations. However, if the signs and symptoms are still present after a considerable period of time, a second intervention might be necessary on the opposite TMJ.

## Competing interests

The authors declare that they have no competing interests.

## Authors’ contributions

EP and AC planned the study and performe the surgical procedure. JGT was responsible for clinical prosthetic dentistry. GHML participated of the literature review. All authors read and approved the final manuscript.

## References

[B1] Nickerson JW Veaco NS Condylotomy in surgery of the temporomandibular joint Oral Maxillofac Surg Clin North Am 1989 1 303 312

[B2] Pullinger AG Monteiro AA History factors associated with symptons of temporomandibular disorders J Oral Rehabil 1988 15 117 124 10.1111/j.1365-2842.1988.tb00760.x 3163728

[B3] Puricelli E **Tratamento cirúrgico da ATM: casos selecionados - Surgical treatment of TMJ: previous cases** Atualização na clínica odontológica: cursos antagônicos 2000 São Paulo: Artes Medicas 479 520

[B4] Lippold C Kruse-Losler B Danesh G Joos U Meyer U Treatment of hemimandibular hyperplasia: The biological basis of condylectomy Br J Oral Maxillofac Surg 2007 45 5 353 360 10.1016/j.bjoms.2006.10.011 17145124

[B5] Campbell W Clinical radiological investigations of the mandibular joints Br J Radiol 1965 38 401 421 10.1259/0007-1285-38-450-401 14301472

[B6] James P The surgical treatment of mandibular joint disorders Ann R Coll Surg Engl 1971 49 310 328 4107180 PMC2388029

[B7] Banks P Mackenzie I Condylotomy. A clinical and experimental appraisal of a surgical technique J Maxillofac Surg 1975 3 170 181 809522 10.1016/s0301-0503(75)80040-3

[B8] Upton LG Sullivan SM The treatment of temporomandibular joint internal derangements using a modified open condylotomy: a preliminary report J Oral Maxillofac Surg 1991 49 578 583 10.1016/0278-2391(91)90338-M 2037913

[B9] Tasanen A Von Konow L Closed condilotomy in the treatment of idiophatic and traumatic pain-dysfunction síndrome of the temporomandibular joint Int J Oral Surg 1973 2 102 106 10.1016/S0300-9785(73)80048-1 4213807

[B10] Machado APB Ataide D Sandri C Vandressen N Jordão J Importancia do raio X e exame fí sico no diagnó stico da artrite psoriá tica e sua prevalencia no Hospital Universitá rio Evangé lico de Curitiba (HUEC) An Bras Dermatol 2005 80 3 345 351

[B11] Harris SR Medlicott MS A systematic review of the effectiveness of exercise, manual therapy, electrotherapy, relaxation training, and biofeedback in the management of temporomandibular disorder Physical Therapy July 2006 86 955 973 16813476

[B12] Senye M Mir CF Morton S Thie NMR Topical nonsteroidal anti-inflammatory medications for treatment of temporomandibular joint degenerative pain: a systematic review J Orofac Pain 2012 26 26 32 22292137

[B13] Banks P The case against mandibular condylotomy in the treatment of the painful, deranged temporomandibular joint J Oral Maxillofac Surg 1996 54 70 74 10.1016/s0278-2391(97)90450-x 9019523

[B14] Hall HD Nickerson JW McKenna SJ Modified condilotomy for treatment of the painful temporomandibular joint with a reducing disc J Oral Maxillofac Surg 1993 51 133 142 10.1016/S0278-2391(10)80009-6 8426252

[B15] Walker RV Kalamanchi S A surgical technique for management of internal derangement of the temporomandibular joint J Oral Maxillofac Surg 1987 45 299 305 10.1016/0278-2391(87)90347-8 3470447

[B16] Albury CD Jr Modified condilotomy for chronic nonreducing disk dislocations Oral Surg Oral Med Oral Pathol Oral Radiol Endod 1997 84 234 240 10.1016/S1079-2104(97)90336-X 9377184

[B17] McKenna SJ Cornella F Gibbs SJ Long-term follow-up of modified condylotomy for internal derangement of the temporomandibular joint Oral Surg Oral Med Oral Pathol 1996 81 509 515 10.1016/S1079-2104(96)80038-2 8734694

[B18] Akinbami BO Evaluation of the mechanism and principles of management of temporomandibular joint dislocation. Systematic review of literature and a proposed new classification of temporomandibular joint dislocation Head Face Med Jun 2011 15 7 10 10.1186/1746-160X-7-10 PMC3127760 21676208

[B19] Upton LG The case for mandibular condylotomy in the treatment of the painful, deranged temporomandibular joint J Oral Maxillofac Surg 1996 54 64 69 10.1016/s0278-2391(97)90449-3 8994470

[B20] Hall HD Werther JR Results of reoperation after failed modified condilotomy J Oral Maxillofac Surg 1997 55 1250 1253 10.1016/S0278-2391(97)90178-6 9371115

[B21] Hall HD Treatment of painful temporomandibular joint dysfunction with the sagittal split ramus osteotomy J Oral Maxillofac Surg 2002 60 1002 1003 10.1053/joms.2002.34407 12215982

